# Alcohol exposure alters pre-mRNA splicing of antiapoptotic Mcl-1L isoform and induces apoptosis in neural progenitors and immature neurons

**DOI:** 10.1038/s41419-019-1673-3

**Published:** 2019-06-06

**Authors:** Martina Donadoni, Stephanie Cicalese, Dipak K. Sarkar, Sulie L. Chang, Ilker Kudret Sariyer

**Affiliations:** 1 Department of Neuroscience, Center for Neurovirology, Temple University Lewis Katz School of Medicine, Philadelphia, PA 19140 USA; 2 The Endocrine Program, Department of Animal Sciences, Rutgers, The State University of New Jersey, 67 Poultry Lane, New Brunswick, NJ 08901 USA; 3 Institute of NeuroImmune Pharmacology and Department of Biological Sciences, Seton Hall University, South Orange, NJ USA

**Keywords:** Cell death in the nervous system, Experimental models of disease

## Abstract

Alternative splicing and expression of splice variants of genes in the brain may lead to the modulation of protein functions, which may ultimately influence behaviors associated with alcohol dependence and neurotoxicity. We recently showed that ethanol exposure can lead to pre-mRNA missplicing of Mcl-1, a pro-survival member of the Bcl-2 family, by downregulating the expression levels of serine/arginine rich splicing factor 1 (SRSF1). Little is known about the physiological expression of these isoforms in neuronal cells and their role in toxicity induced by alcohol exposure during the developmental period. In order to investigate the impact of alcohol exposure on alternative splicing of Mcl-1 pre-mRNA and its role in neurotoxicity, we developed a unique primary human neuronal culture model where neurospheres (hNSPs), neural progenitors (hNPCs), immature neurons, and mature neurons were cultured from the matching donor fetal brain tissues. Our data suggest that neural progenitors and immature neurons are highly sensitive to the toxic effects of ethanol, while mature neuron cultures showed resistance to ethanol exposure. Further analysis of Mcl-1 pre-mRNA alternative splicing by semi-quantitative and quantitative analysis revealed that ethanol exposure causes a significant decrease in Mcl-1L/Mcl-1S ratio in a dose and time dependent manner in neural progenitors. Interestingly, ectopic expression of Mcl-1L isoform in neural progenitors was able to recover the viability loss and apoptosis induced by alcohol exposure. Altogether, these observations suggest that alternative splicing of Mcl-1 may play a crucial role in neurotoxicity associated with alcohol exposure in the developing fetal brain.

## Introduction

In the US about 10.2% women reports EtOH consumption with 3.1% reporting binge drinking during pregnancy^1–3^. Individuals with fetal alcohol exposure (FAE) possess developmental delays along with cognitive and behavioral impairments^3–6^. The most devastating and extreme consequence of fetal EtOH exposure is the neuronal loss due to the exacerbation of selective programmed neuronal death, which is a normal aspect of CNS development during developmental organogenesis. Excessive neuronal death disrupts the development of normal neural networks and may lead to structural changes along with cognitive and behavioral dysfunctions^7,8^. The behavioral abnormalities associated with excessive neuronal death include generalized impairment in the development of motor competence (cerebellar abnormalities)^9,10^, learning and memory deficits (hippocampal abnormalities)^11–13^, and stress hyperresponse and anxiety (hypothalamic abnormalities)^14–16^. The cellular mechanisms involved in increased neuronal death leading to altered structural changes and behavioral functions in fetal EtOH exposed offspring are not clearly demonstrated.

In addition to transcription, alternative pre-mRNA splicing is a key cellular process whereby utilization of potential splice sites of the pre-mRNA in various combinations by spliceosome under the regulation of alternative splicing factors makes a significant contribution to proteomic diversity by leading to expression of various isoforms of the proteins with distinct functions^17,18^. Recent studies suggest that alternative pre-mRNA splicing of genes may contribute to the development of alcohol-induced disorders^19–23^. However, little is known regarding the potential impact of alcohol on alternative splicing of genes during the fetal development and its involvement in the development of fetal alcohol spectrum disorder (FASD). We have recently shown that EtOH exposure of fetal neurons suppresses expression levels of serine/arginine rich splicing factor 1 (SRSF1) and causes missplicing of myeloid cell leukemia 1 (Mcl-1) by favoring the Mcl-1S splicing over Mcl-1L^24^. Several studies suggested that while the longer gene product Mcl-1L enhances cell survival by inhibiting apoptosis in response to various stress conditions in different cell types, the alternatively spliced shorter gene product Mcl-1S may promote apoptosis^25,26^. Hence, the possibility arises that alternative splicing of Mcl-1 may be involved in the mechanism involved in ethanol-induced neurotoxicity during the developmental period. In order to determine this, we investigated the potential impact of EtOH exposure on cellular toxicity and alternative splicing of Mcl-1 pre-mRNA and its involvement with cytotoxicity induced by EtOH in different lineages of neuronal cultures in an in vitro primary culture model where human neurospheres, neural progenitors, immature neurons, and mature neuron cultures were prepared and utilized from matching human fetal brain tissue. Our results suggest that human neurospheres, neural progenitors, and immature neurons but not the mature neurons are highly sensitive to the toxic effects of EtOH in a time and dose dependent manner. Interestingly, Mcl-1 missplicing leading to a decrease in Mcl-1L/Mcl-1S ratio is mainly observed in neural progenitors and immature neurons with no significant alteration in mature neurons. More interestingly, overexpression of antiapoptotic Mcl-1L isoform in neural progenitor cells is able to reverse the viability loss and apoptosis induced by EtOH.

## Results

### Primary human neuronal cultures as a model to study alcohol effects

**Fig. 1:**
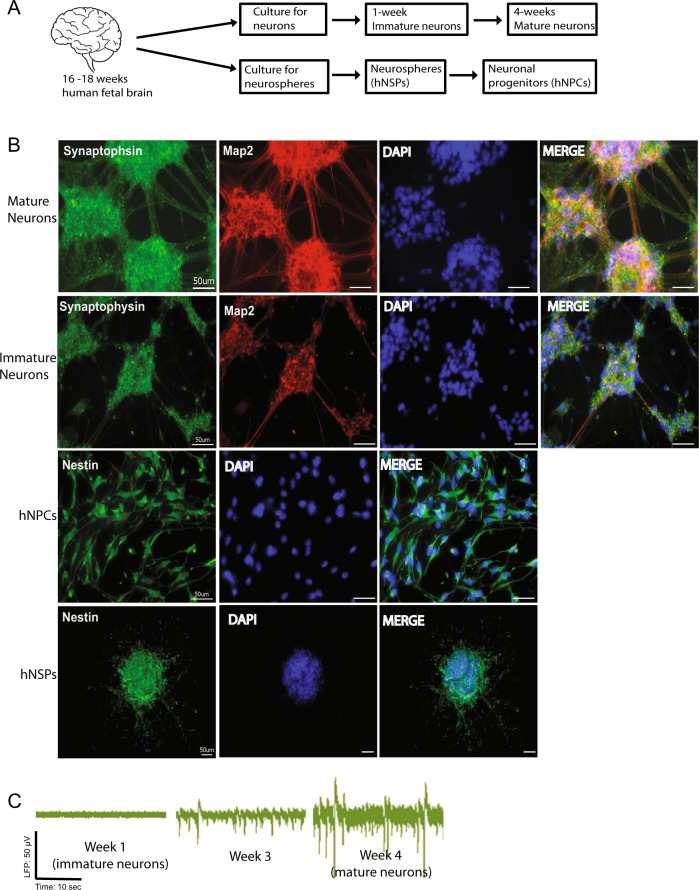
Characterization of primary human neuronal culture model. Primary human fetal neuronal cells (PHFNs) were cultured from matching human fetal donor brains (16–18 weeks of gestation). **a** Schematic presentation of human immature neurons (1 week in culture), mature neurons (4 weeks in culture), neurospheres (hNSPs), and hNSPs-derived neural progenitors (hNPCs). **b** Immature neurons, mature neurons, hNSPs and hNPCs were plated in chamber slides, fixed, and immunostained for Nestin (green; hNSPs and hNPCs), and Synaptophysin (green) and Map2 (red) co-staining (immature and mature neurons). Cells nuclei were also counterstained with DAPI (blue). **c** hNPCs were plated in neural differentiation media and local field potentials (LFP) were tested at 1, 3, and 4 weeks post differentiation by multielectrode arrays (MEA). hNPCs-derived neurons at 1 week were called immature neurons with no LFP recordings and 4 weeks cultures were classified as mature-functional neurons with high LFP recordings. Scale bar represent 50 μM

We have developed a unique primary human culture model for neuronal cells to study the possible impact of EtOH on neuronal viability at different stages of differentiation (Fig. 1a). Primary human fetal neurons (PHFNs) and neurospheres (hNSPs) were cultured from matching human fetal donor brains (16–18 weeks of gestation) as described in materials and methods. In parallel to direct neuron cultures, hNSPs were also established from matching donor brains and maintained in the culture. In order to establish neural progenitors, neurospheres were dissociated and cultured as monolayers of neuronal progenitors (hNPCs) as described in materials and methods. Immunocytochemical verification revealed that young and mature neurons were stained for synaptophysin and Map2, hNSPs and hNPCs were stained for Nestin (Fig. 1b). PHFN cultures were followed for neuronal action potentials as the criteria for their full differentiation by multielectrode array (MEA) studies measuring local field potentials (LFP) at week 1, 3, and 4 post culturing. As shown in Fig. 1c, PHFNs started to show LFP activity at 3 weeks and reached the peak levels at 4 weeks post culturing. Neuronal cultures at 1 week were called immature neurons with minimal LFP recordings and 4 weeks cultures were named mature (functional) neurons with maximum LFP recordings.

### EtOH induces apoptosis in neural progenitors and immature neurons but not in mature neurons in primary cultures

**Fig. 2:**
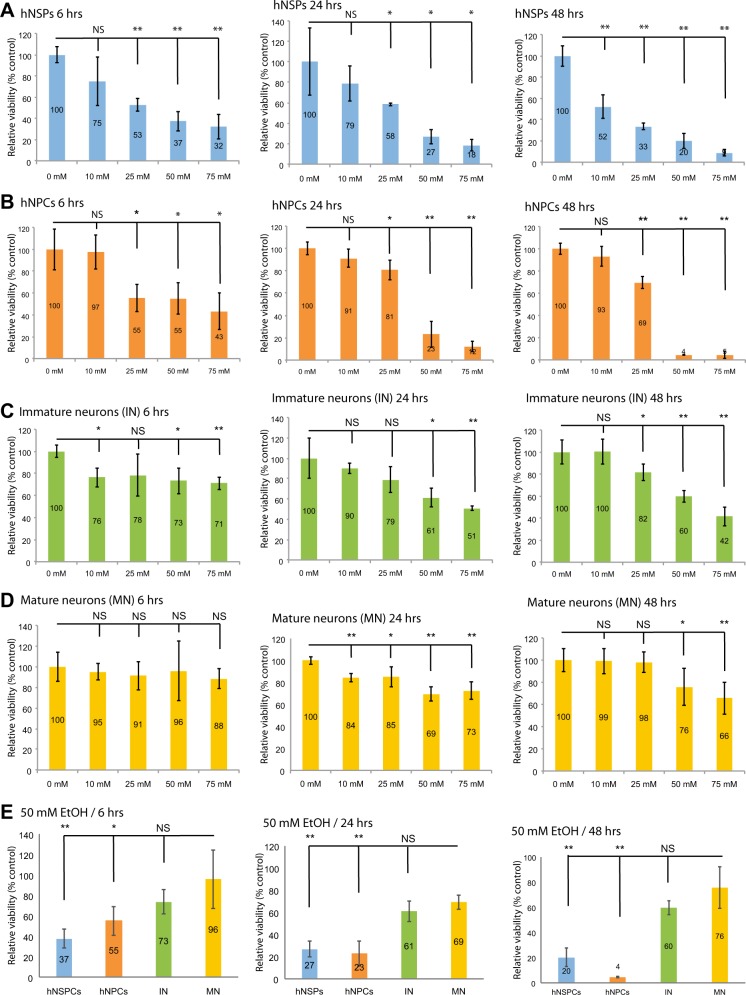
Effect of EtOH exposure on viability of neuronal cells at different stages of differentiation. hNSPs (**a**), hNPCs (**b**), immature neurons (**c**) and mature neurons (**d**) were plated in 12-well culture dishes. Cells were exposed to EtOH at 0, 10, 25, 50, and 75 mM concentrations for 6, 24, and 48 h. Cellular viability was assessed at each time point by MTT assay. **e** Relative cell viability (% control-untreated) were also compared for hNSPs, hNPCs, immature neurons (IN), and mature neurons (MN) exposed to 50 mM EtOH at 6, 24, and 48 h post exposures. Data are mean + SEM of three independent replicates. “*P”* values were calculated in comparison with control-untreated cells (**a**–**d**) or with mature neurons exposed to 50 mM EtOH (**e**). *p < 0.05, **p < 0.001, NS no significant difference (via *t* test)

Next, we investigated the impact of EtOH exposure on viability of neuronal cells at different lineages. hNSPs, hNPCs, immature neurons (1-week differentiation with no LFP activity), and mature neurons (4-weeks differentiation with high LFP activity) were established as explained above (Fig. 1). Cells were exposed to EtOH at 0, 10, 25, 50, and 75 mM concentrations. To maintain constant ethanol concentrations in cell culture media, we utilized an “anti-evaporation system” as described before^27,28^. Metabolic activities of the cultures were monitored by an MTT cell viability assay at 6, 24, and 48 h post exposures. As shown in Fig. 2a, cell viability responses of hNSPs were highly sensitive to EtOH as early as 6 h post exposure at 25 mM and higher concentrations. The effect was more dramatic for the 24 and 48 h post exposures and nearly 80–90% of the cells lost their viability at 50 and 75 mm concentrations for 24 and 48 h post exposures. hNPCs also showed reduced metabolic rates and loss of cell viability with a significant decrease at 25, 50, and 75 mM concentrations at 6, 24 or 48 h (Fig. 2b). The effect was highly dramatic for the 24 and 48 h post exposures at 50 and 75 mM concentrations. Immature neuron cultures showed a significant cell viability loss at 6 and 24 h post exposures mainly at 50 and 75 mM EtOH concentrations and 25 mM and higher concentrations at 48 h post exposures (Fig. 2c). Interestingly, mature neuron cultures were resistant to the toxic effects of EtOH for the initial 6 h post treatments up to 75 mM concentrations. Mature neuron cultures showed low to moderate but significant viability loss at 10 to 75 mM concentrations at 24 h post exposures. Interestingly, viability loss was recovered with no significant difference of control neurons than neurons treated at 10 and 25 mM concentrations for 48 h. On the other hand, mature neurons showed a moderate but significant viability loss at 50 and 75 mM EtOH treatments for 48 h post treatments compared to neural progenitors and immature neurons. Differences in cellular metabolic activities and viability between mature neuron cultures and neurospheres, progenitors, and immature neurons were also analyzed and compared at 6, 24, and 48 h post treatments for the 50 mM EtOH exposure. The results suggest that the difference in metabolic activity and cellular viability reduction induced by EtOH exposure was quite significant between mature neurons and neural progenitors as well as neurospheres (Fig. 2e). On the other hand, the reduction rate of viability loss in immature neurons was not significantly different than those for the mature neuron cultures exposed to 50 mM EtOH up to 48 h post treatments.

**Fig. 3:**
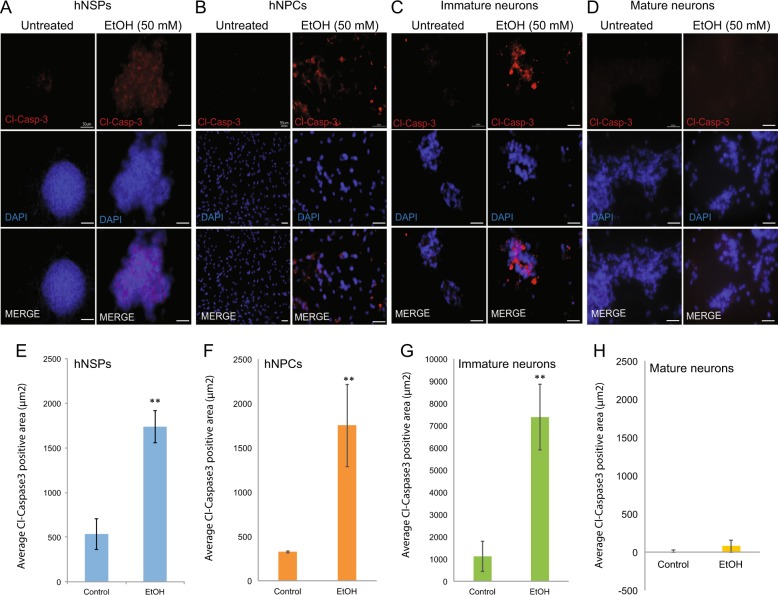
EtOH exposure induces cleaved caspase-3 activation in neurospheres, neural progenitors and immature neurons, but not in fully differentiated mature neurons in primary cultures. hNSPs (**a**, **e**), hNPCs (**b**, **f**), immature neurons (**c**, **g**), and mature neurons (**d**, **h**) were isolated and cultured from matching human fetal brain in chamber slides. Cells were either treated or untreated with EtOH (50 mM) for 24 h, fixed, and processed for immunocytochemical determination of cleaved caspase-3 protein. Nuclei were also counterstained with DAPI. Cleaved caspase-3 activation was quantified as cl-caspase3 positive area (μm2) based on red fluorescein and presented as bar graph from three independent replicates. Data are mean + SEM of three independent replicates. *p < 0.05, **p < 0.001 (via *t* test). Scale bar represent 50 μM

Although MTT assay can measure cytotoxicity (loss of viable cells) of cultured cells by assessing cell metabolic activity, it may also potentially suggest cytostatic activity (shift from proliferation to quiescence) of cells induced by EtOH exposure. To gain more insight into EtOH-mediated cytotoxicity in different lineages of neuronal cells, hNSPs, hNPCs, immature neurons, and mature neurons were also plated in chamber slides, treated with EtOH (50 mM) for 24 h, fixed and processed by immunocytochemistry for cleaved caspase-3, an apoptosis marker. As shown in Fig. 3a, e, EtOH exposure had a significant impact on the morphology of hNPCs with a robust cleaved caspase-3 induction. Similarly, neural progenitors (hNPCs) derived from hNSPs (Fig. 3b, f) and immature neurons (Fig. 3c, g) were also very sensitive to EtOH treatment with an extensive cleaved caspase-3 activation. Interestingly, mature neuronal cultures had no visible sign of cellular toxicity and cleaved caspase-3 activation (Fig. 3d, h). These results suggest that while neural progenitors and immature neurons are highly sensitive, mature neurons show resistance to the neurotoxic effects of EtOH.

### EtOH-mediated missplicing of Mcl-1 pre-mRNA is preferentially induced in neural progenitors and immature neurons

**Fig. 4:**
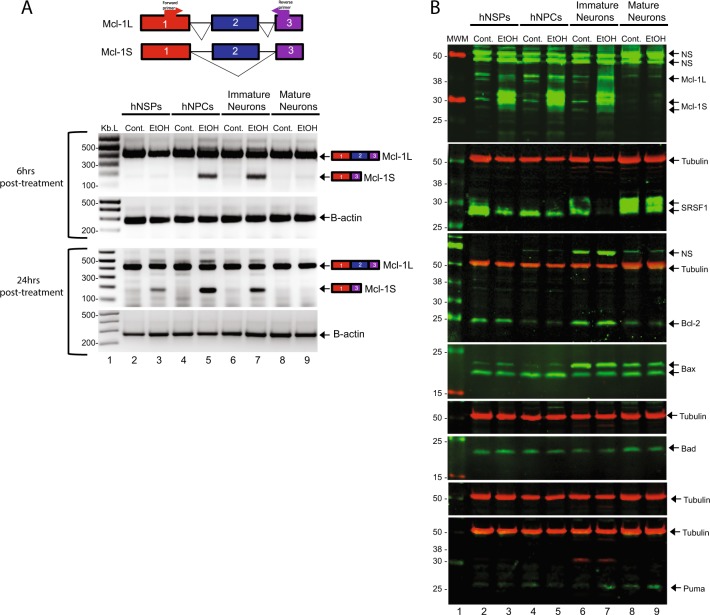
EtOH exposure leads to alternative splicing of Mcl-1S isoform in neurospheres, neural progenitors, and immature neurons but not in mature neurons. **a** hNSPs, hNPCs, immature neurons, and mature neurons were isolated and cultured from matching human fetal brain and exposed to 50 mM EtOH. Total RNA was isolated from control cells (cont.) and from cells exposed to EtOH by using a commercial RNA extraction kit at 6 and 24 h post exposures. One microgram of total RNA was used in reverse transcription reactions and cDNA was synthesized. Splicing isoforms of Mcl-1 were analyzed by RT-PCR using specific primers at 6 and 24 h post exposures, and separated and monitored on agarose gel by ethidium bromide staining. The location of primer pairs and map of exon-intron structure of the Mcl-1 pre-mRNA are schematized on the top scheme. **b** hNSPs, hNPCs, immature neurons, and mature neurons were isolated and cultured from matching human fetal brain and exposed to 50 mM EtOH. Whole cell protein lysates were isolated from control cells (untreated-cont.) and from cells exposed to 50 mM EtOH for 24 h. Western blots of protein lysates were performed to access the expression levels of Mcl-1L, Mcl-1S, SRSF1, Bcl-2, Bax, Bad, and Puma. Tubulin was probed in the same blots as loading control. NS depicts “Nonspecific” band recognized by the antibodies

To gain insight into possible impact of EtOH on alternative splicing of Mcl-1, alternative splicing of Mcl-1 pre-mRNA was further analyzed in different lineages of neuronal cells. The cultures of hNSPs, hNPCs, immature neurons, and mature neurons were exposed to EtOH (50 mM) for 6 and 24 h. Total RNA from cells was isolated and analyzed by RT-PCR for amplification and detection of Mcl-1 long and short isoforms. The antiapoptotic isoform Mcl-1L is expressed in hNSPs, hNPCs, immature neurons, and mature neurons (Fig. 4a). Interestingly, consistent with cell viability and apoptosis assays (Figs. 2 and 3, respectively), proapoptotic isoform Mcl-1S is only induced in neuronal progenitors and immature neurons at 6 and 24 h post exposures (Fig. 4a). On the other hand, induction of Mcl-1S isoform in hNSPs was only observed at 24 h post exposures. EtOH exposure did not alter the splicing of Mcl-1 pre-mRNA in mature neuron cultures at both 6 and 24 h post exposures. These results suggest that EtOH exposure can selectively induce alternative splicing of Mcl-1 mRNA in neural progenitors and immature neurons. In order to confirm translation of Mcl-1S mRNA induced by EtOH and possible impact of EtOH on expression of splicing regulatory protein SRSF1 and other members of Bcl-2-associated genes, including Bcl-2, Bax, Bad, and Puma, whole cell protein lysates obtained from hNSPs, hNPCs, immature neurons, and mature neurons exposed to EtOH (50 mM) for 24 h were processed by western blotting (Fig. 4b). Consistent with alternative splicing of Mcl-1S mRNA, EtOH exposure induced Mcl-1S expression in hNSPs, hNPCs, and immature neurons, but not in mature neuron cultures. Interestingly, Mcl-1L expression was quite low in control-untreated cells with slight reduction in their expression in hNSPs and hNPCs and no visible change in immature and mature neurons at 24 h post treatments. These differences in Mcl-1L expression could be related to the differences in its stability or half-life across the cell types. In line with our previous report^24^, EtOH exposure caused a dramatic reduction in SRSF1 protein levels in hNSPs, hNPCs, and immature neurons. Interestingly, SRSF1 levels in mature neuron cultures were slightly reduced but maintained in cells exposed to EtOH suggesting that its expression levels were sufficient to maintain Mcl-1L splicing over Mcl-1S. The expression levels of other members of Bcl-2-associated genes, including Bcl-2, Bax, Bad, and Puma were not significantly altered by EtOH exposure in all the cells types analyzed.

### Quantitative analysis of Mcl-1L/Mcl-1S ratio in neural cells exposed to EtOH

**Fig. 5:**
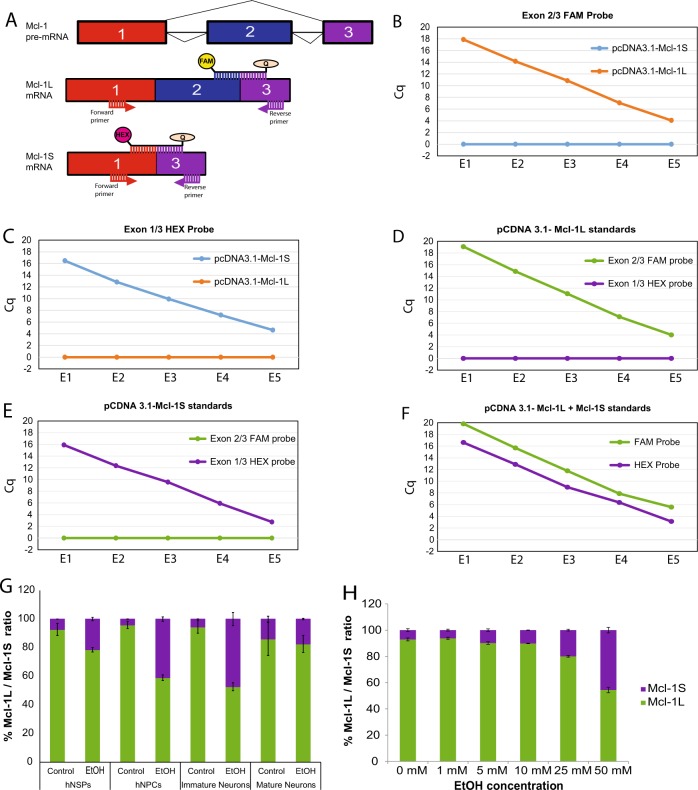
Real-time qRT-PCR of Mcl-1L and Mcl-1S isoforms in response to EtOH exposure in neuronal cells. **a** Schematic representation of Mcl-1L probe (spanning exons 2/3 junction) with a FAM fluorophore and MCL-1S probe (spanning exons 1/3 junction) with a HEX fluorophore. B. Specificity of Mcl-1L-FAM (**b**) and Mcl-1S-HEX (**c**) probes were analyzed by Q-PCR utilizing expression vectors encoding Mcl-1L and Mcl-1S isoforms as standards. Sensitivity of both probes was determined in dual probe reactions in the presence of both Mcl-1L (**d**) or Mcl-1S standards (**e**) by Q-PCR. Quantitative amplification and differential detection of both isoforms were also analyzed in a single reaction in the presence of both standards (**f**). E1–E5 depicts the log scale of plasmid copy numbers. Cq represents the quantitative PCR cycle in which fluorescence can be detected. **g** hNSPs, hNPCs, immature neurons, and mature neurons were exposed to EtOH (50 mM) for 24 h. Total RNA was extracted and processed by real-time qRT-PCR by utilizing Mcl-1L probe for exon 2/3 junction with FAM fluorophore and Mcl-1S probe for exon 1/3 junction with HEX fluorophore. Percent of Mcl-1L/Mcl-1S ratio was presented as bar graph from three independent assays. **h** hNPCs were exposed to increasing concentrations of EtOH for 24 h. Total RNA was extracted and processed by real-time qRT-PCR by utilizing Mcl-1L-FAM and Mcl-1S-HEX probes. Percent of Mcl-1L/Mcl-1S ratio was presented as bar graph. Data are mean + SEM of three independent replicates

The RT-PCR assay provided semi-quantitative analysis of long (L) and short (S) isoforms of Mcl-1 pre-mRNA. In order to quantify the alternative splicing of both isoforms in control and EtOH exposed neuronal cells, we developed a unique real-time qRT-PCR based assay by utilizing probes specifically designed at exon junctions of Mcl-1 isoforms and two fluorophores with different excitation/emission spectrum. Mcl-1L probe was designed for the exons 2/3 junction with a FAM fluorophore and Mcl-1S probe was designed for exons 1/3 junction with a HEX fluorophore (Fig. 5a). Multiplexing approach was taken to amplify, distinguish, and quantify Mcl-1L and Mcl-1S isoforms in a single reaction. Mcl-1L and Mcl-1S isoforms were cloned into a mammalian expression vector and utilized as standards. The specificity of each probe was first established in a single probe reaction in the presence of both standards for Mcl-1L and Mcl-1S isoforms. As shown in Fig. 5b, c, each probe showed differential and quantitative detection of both isoforms with FAM-probe detecting Mcl-1L isoform and HEX-probe detecting Mcl-1S isoform. Next, sensitivity of both probes was determined in dual probe reactions in the presence of both Mcl-1L and Mcl-1S standards. As shown in Fig. 5d, e, both Mcl-1L and Mcl-1S isoforms were specifically and selectively recognized by FAM and HEX-probes, respectively. Finally, quantitative amplification and differential detection of both isoforms were tested and confirmed in a single reaction in the presence of both Mcl-1L and Mcl-1S standards (Fig. 5f). Once the real-time qRT-PCR assay was established, the impact of EtOH exposure on alternative splicing of Mcl-1L and Mcl-1S isoforms was analyzed in different lineages of neuronal cells. hNSPs, hNPCs, immature neurons, and mature neurons were exposed to EtOH (50 mM) and total RNA extracts were processed by real-time qRT-PCR at 24 h post exposures. Consistent with semi-quantitative RT-PCR analysis (Fig. 4), EtOH exposure caused a significant shift in Mcl-1L and Mcl-1S ratio by favoring Mcl-1S alternative splicing over Mcl-1L in hNSPs, hNPCs, and immature neurons with no significant alteration in mature neurons in cultures. Moreover, dose response effects of EtOH on alternative splicing of Mcl-1 pre-mRNA were also analyzed by real-time qRT-PCR in neural progenitors. hNPCs were exposed to EtOH at 0, 1, 5, 10, 25, and 50 mM concentrations and total RNA extracts were processed by real-time qRT-PCR at 24 h post exposure for the selective and quantitative analysis of Mcl-1L and Mcl-1S isoforms. As shown in Fig. 5h, EtOH caused a significant decrease in Mcl-1L / Mcl-1S ratio at 25 mM and 50 mM concentrations in a dose dependent manner.

**Fig. 6:**
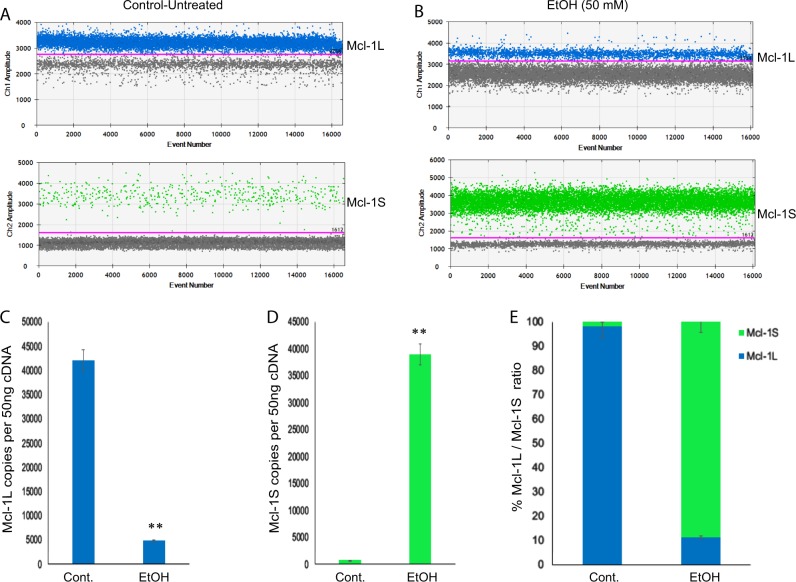
Droplet digital-PCR analysis of Mcl-1L and Mcl-1S isoforms in hNPCs exposed to EtOH. **a**, **b** Representative ddPCR reads of cDNA samples from untreated (**a**) and EtOH treated (**b**) hNPCs for Mcl-1L (FAM) and Mcl-1S (HEX) transcript copies. **c** ddPCR reads of Mcl-1L copies from untreated and EtOH treated cells were represented as bar graph. **d** ddPCR reads of Mcl-1S copies from untreated and EtOH treated cells were represented as bar graph. **e** Percent of Mcl-1L/Mcl-1S copies was presented as bar graph

RT-PCR and RT-qPCR have been the major standard assays for the relative quantification of alternatively spliced protein isoforms for a decade. With the advance of technology, droplet digital PCR (ddPCR) is becoming widely implemented for these types of analysis due to the high-precision, absolute quantification of nucleic acid target sequences with absolute quantities without the need for standard curves^29,30^. To gain more insights into the expression of Mcl-1L and Mcl-1S isoforms in neural progenitors, cDNA samples from hNPCs either exposed to EtOH (50 mM for 24 h) or control-untreated cells were processed by ddPCR utilizing a pair of differential primers and a set of probes for each isoform as FAM-probe detecting Mcl-1L isoform and HEX-probe detecting Mcl-1S isoform in a single reaction. As shown in Fig. 6a–d, untreated hNPCs mainly express Mcl-1L isoform with less than 10% Mcl-1S mRNA copies. On the other hand, consistent with RT-PCR and qRT-PCR analysis, EtOH caused a shift in Mcl-1L / Mcl-1S ratio by favoring Mcl-1S splicing over Mcl-1L (Fig. 6e). Interestingly, when the true copies of each isoform transcripts were determined and compared by ddPCR, the influence of EtOH on induction of Mcl-1S and reduction of Mcl-1L isoform splicing was even more dramatic than what it was suggested by RT-PCR and qRT-PCR analysis (compare Fig. 5 and Fig. 6).

### Ectopic expression of Mcl-1L isoform prevents EtOH induced toxicity in hNPCs

**Fig. 7:**
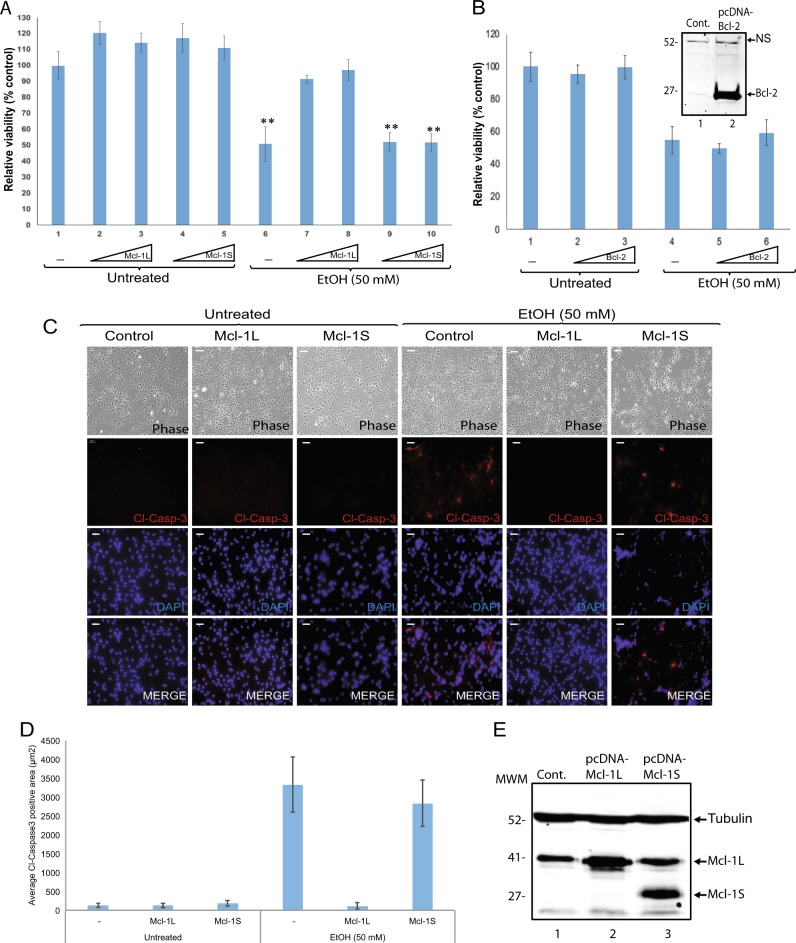
Ectopic expression of Mcl-1L isoform is protective against toxic effects of EtOH in neural progenitors. **a** hNPCs were transiently transfected with increasing concentration of expression vectors encoding Mcl-1L or Mcl-1S isoforms. At 24 h post transfections, cells were either exposed to EtOH (50 mM) or left untreated. At 24 h post EtOH exposure, cell viability was assessed by MTT assay, normalized to control (lane 1), and shown as bar graph. **b** hNPCs were transiently transfected with increasing concentration of an expression vector encoding human Bcl-2. At 24 h post transfections, cells were either exposed to EtOH (50 mM) or left untreated. At 24 h post EtOH exposures, cell viability was assessed by MTT assay, normalized to control (lane 1), and shown as bar graph. Whole cell protein lysates from hNPCs either untransfected (cont.) or transfected with an expression vector encoding human Bcl-2 were processed by western blotting using anti-Bcl-2 antibody and shown as integrated into the bar graph. NS depicts “Nonspecific” band recognized by the antibody. **c** hNPCs were transiently transfected with expression vectors encoding Mcl-1L or Mcl-1S isoforms. At 24 h post transfections, cells were either exposed to EtOH (50 mM) or left untreated. At 24 h post EtOH exposure cells were fixed and processed by immunocytochemistry for cleaved caspase-3 immunostaining. Nuclei were also counterstained with DAPI. **d** Cleaved caspase-3 activation was quantified as cl-caspase3 positive area (μm2) based on red fluorescein and presented as bar graph from three independent replicates. Data are mean + SEM of three independent replicates. **p < 0.001 (via *t* test). Scale bar represent 50 μM. **e** Whole cell protein lysates from hNPCs either untransfected (cont.) or transfected with expression vectors encoding Mcl-1L or Mcl-1S isoforms were processed by western blotting using anti-Mcl-1 and anti-Tubulin antibodies. Data are mean + SEM of three independent replicates. *P* values were calculated in comparison with untreated cells (0 mM). *p < 0.05, **p < 0.001 (via *t* test). Scale bar represents 50 μM

In addition, we conducted studies to investigate potential blockade of toxicity associated with EtOH exposure by overexpression of Mcl-1L isoform. Neuronal progenitor cells were transfected with mammalian expression vectors encoding Mcl-1L or Mcl-1S isoforms and either exposed to 50 mM EtOH or left untreated. Cell viability were analyzed by MTT cell viability assay. As shown in Fig. 7, EtOH exposure caused a significant reduction in cellular viability (Fig. 7a, b), altered morphology (Fig. 7c), and induced cleaved caspase-3 activation (Fig. 7d) in neural progenitors. Interestingly, ectopic expression of Mcl-1L isoform showed a significant recovery of cellular morphology, viability, and cleaved caspase-3 activation suggesting that EtOH-mediated cellular toxicity was associated with the reduced levels of Mcl-1L isoforms in neural progenitors. On the other hand, ectopic expression of Mcl-IS did not affect cell viability or cleaved caspase-3 in ethanol-activated hNPCs cells. Overexpression of Mcl-1S did not show any significant impact on cellular viability and apoptosis in control-untreated cells. These results suggest that EtOH-mediated toxicity may not be associated with increased copies of Mcl-1S isoform, but may be related to the decreased copies of Mcl-1L in neural progenitor cells. In order to determine specificity of Mcl-1L in rescue of cytotoxicity induced by EtOH, possible impact of Bcl-2, a key member of BCL2 family genes, was also assessed (Fig. 7b). Neural progenitor cells were transfected with increasing concentrations of a mammalian expression vector encoding human Bcl-2 and either exposed to 50 mM EtOH for 24 h or left untreated. Cell viability was analyzed by MTT cell viability assay. The results suggest that unlike Mcl-1L, Bcl-2 overexpression had no significant rescue of viability loss induced by EtOH, suggesting a unique and specific role of Mcl-1 gene in EtOH-mediated cytotoxicity in neural progenitors.

## Discussion

Herein, by qualitative and quantitative analysis, we report that alcohol exposure selectively induces a shift in Mcl-1L/Mcl-1S ratio in neural progenitors and immature neurons but not in mature neuron cultures. Interestingly, the reduction in Mcl-1L and increase in Mcl-1S isoforms correlates with viability loss and cell death due to the apoptosis activation in neural progenitors. Furthermore, ectopic expression of Mcl-1L isoform is able to recover toxicity induced by EtOH suggesting a novel role of Mcl-1L isoform in neurotoxicity associated with EtOH exposure.

**Fig. 8:**
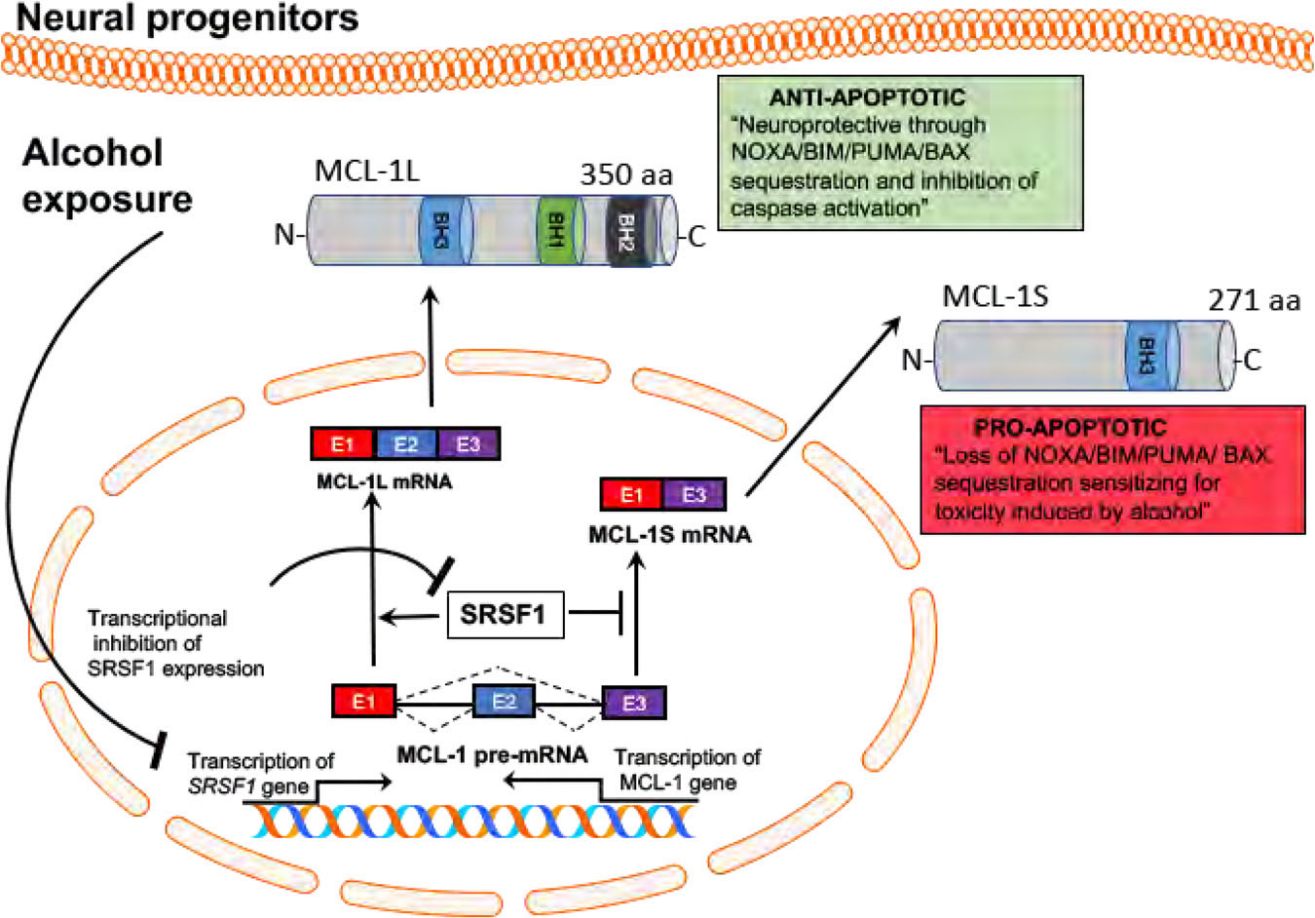
Schematic representation of proposed model of Mcl-1 pre-mRNA missplicing under the influence of alcohol exposure leading to sensitization of neural progenitors and immature neurons for the toxic effects of alcohol. EtOH exposure in neural progenitors leads to transcriptional suppression of SRSF1^24,41^ that leads to a shift in Mcl-1S splicing over Mcl-1L isoform with exon 2 exclusion resulting reduced copies of Mcl-1L transcript. Compared to Mcl-1L, Mcl-1S lacks the BH1 and BH2 domains which are critical for interacting and sequestering proapoptotic genes, such as NOXO, BIM, PUMA, and BAX, for the inhibition of apoptotic stimulus induced by EtOH

Mcl-1 was first discovered in myeloid leukemia cells during differential screening of cDNA libraries^31^. Mcl-1 contains at least 3 putative Bcl-2 homology (BH) domains and experimentally can protect against apoptosis by interacting and sequestering proapoptotic Bcl-2 family member proteins (Fig. 8) including, Noxa (a BH3 only member of Bcl-2 family), PUMA (apoptosis regulator induced by p53), Bim (Bcl-2-like protein 11), and Bax protein (Bcl-2 associated X)^32–34^. Overexpression of Mcl-1 has been seen in various human tumors, including hematologic leukemia, ovarian cancer, prostate cancer, lung cancer, breast cancer, and pancreatic cancers^35–38^. Downregulation of Mcl-1 expression in tumor cells has been shown to increases the cancer cell sensitivity to drug treatments^39,40^, suggesting that Mcl-1 may play a critical role in cellular viability. Alternative splicing of Mcl-1 gene to form Mcl-1L and Mcl-1S isoforms is mainly regulated by SRSF1^24,41^ (Fig. 8). The shorter isoform Mcl-1S lacks BH1 and BH2 domains due to the exclusion of exon 2 during splicing. While Mcl-1L could enhance cell survival, the alternatively spliced Mcl-1S was shown to be associated with apoptosis induction^26,42^. In addition to its role in the regulation of apoptosis, Mcl-1 plays a key role in nervous system development and neuronal cell death. Neuronal precursors within the ventricular zone and newly committed neurons in the cortex are shown to express high levels of Mcl-1^43^ and Mcl-1 upregulation has been shown to have a role in survival of neuronal precursor cells^44^. Here we also show that EtOH-mediated reduction in Mcl-1L and increase in Mcl-1S isoforms lead to the activation of apoptosis and cell death in neural progenitors. In addition, overexpression of Mcl-1L isoform can prevent ethanol-induced cell death. On the other hand, overexpression of Mcl-1S isoform in neural progenitors was not sufficient to induce apoptosis or cell toxicity, suggesting that Mcl-1S may be the ineffective form of Mcl-1L. These results suggest that Mcl-1L is likely the key player in the mechanism of increased susceptibility of neural progenitors to the toxic effects of EtOH.

In our study, we found that human neural progenitors and immature neurons were highly sensitive to the toxic effects of EtOH. Exposure to EtOH at various concentrations led to a significant reduction in metabolic activity and viability of these cells and resulted in activation of caspase-3 and induction of apoptosis. On the other hand, although a modest but significant reduction in metabolic activity and viability was observed, there was no significant caspase-3 activation in mature neuron cultures isolated from the matching fetal brain suggesting that while neural progenitors and immature neurons are highly sensitive to the toxic effects of EtOH, mature neurons may possess resistance at the same concentrations. These observations are in line with studies where the toxic effects of EtOH were also described on neural stem cells and progenitors during adult neurogenesis in in vitro cell culture and in vivo experimental animal models^45–48^. In our neuronal culture model, alcohol toxicity assessments by cell viability and apoptosis analysis showed a robust toxicity in neural progenitor cells with only modest effect in neurosphere cultures. This could be due to the nature of these cultures been growing in suspension as spheres rather than monolayers on the surface of the plates as progenitors do. As one would expect, EtOH may not diffuse efficiently and reach the inner layers of neurospheres due to the growth characteristics of these cells. In fact, immunocytochemical analysis of caspase-3 activation suggest that while the outer layer of neurospheres showed higher cleaved caspase-3 activation, inner layers had fewer response to apoptosis activation. Another challenge in this type of studies is the maintenance of same number of cells from different cell types in culture during the duration of assays. While immature and mature neurons are post mitotic, neurospheres and progenitors grow and replicate. We believe that some of the differences in the levels of Mcl-1S alternative splicing and sensitivity to the EtOH across cell types could be explained by differences in cell characteristics.

Collectively, our data suggests that EtOH exposure causes viability loss and apoptosis in neural progenitors and immature neurons with a limited impact on fully differentiated mature neuron cultures, suggesting a possible mechanism by which prenatal alcohol exposure exerts detrimental deficits in the brain. In addition, selective missplicing of Mcl-1 is associated with neural progenitor cell death and might be the underlying mechanism of increased sensitivity of progenitors to the toxic effects of EtOH. Altogether, these results suggest that EtOH-mediated missplicing of Mcl-1 pre-mRNA may contribute to FASD phenotype due to the depletion of neural progenitors in the developing fetal brain.

## Materials and methods

### Ethics statement

All samples were obtained and utilized in accordance with Temple University Human Subjects Protections and the approval of the institutional review board.

### Primary human fetal neuronal cultures

Human fetal neuron cultures and neurospheres (hNSPs) were obtained from the Basic Science Core I of the Comprehensive NeuroAIDS Center (CNAC)– headed by Dr. Kamel Khalili at Temple University Lewis Katz School of Medicine. hNSPs were cultured and maintained in DMEM/F12 media supplemented with N2 supplement, FGF, Glutamax, pen/strep, and Hepes (5mM). hNSPs were expanded and cryopreserved for future expansion and usage. Neural progenitors (NPCs) were cultured from hNSPs as follows: hNSPs were dissociated by trypsin, filtered through cell strainers (100 mic), and plated on Geltrex coated dishes in NPC differentiation media consist of neurobasal media supplemented with B27, FGF, glutamax, Na-pyruvate, N-acetyl-L-cysteine, and pen/strep. hNPCs cultures were expanded and cryopreserved for future expansion and usage. hNPCs and hNSPs were characterized by immunocytochemical analysis of GFAP, Nestin, and Sox2 staining. Immature and mature neuron cultures were prepared directly from the matching fetal brains by differentiation. Fetal human brain cells were plated in culture dishes coated with laminin and Poly-L-ornitine. Cells were differentiated into neurons by neuronal differentiation media consist of neurobasal media supplemented with B27, glutamax, BDNF, GDNF, and pen/strep for 1-week for immature neuron cultures and 4-weeks for mature neuron cultures. Immature and mature neurons were characterized by Map2 and synaptophysin immunostaining. Neuronal differentiation and function were monitored electrophysiologically by multielectrode arrays (MEA) measuring local field potentials (LFP) at weeks 1, 2, 3, and 4 post cultures.

### Plasmid constructs

pcDNA3.1-Mcl-1L plasmid encoding human Mcl-1L isoform (NCBI Ref: NM_021960.4) was reported previously^49^ and obtained from Addgene (#25375; Cambridge, MA). pcDNA3-Bcl-2 plasmid encoding human Bcl2 was reported previously^50^ and obtained from Addgene (#19279; Cambridge, MA). Mcl-1S isoform (NCBI Ref: NM_182763.2) was cloned into the eukaryotic expression vector pcDNA3.1(+) at EcoRI/XhoI restriction enzyme sites and labeled as pcDNA3.1-Mcl-1S. The plasmid was created with the following primers: hMcl-1S-EcoRI-F: 5′-CCCTCGTAAAGAATTCATGTTTGGCCTCAAAAGAAA-3′ and hMcl-1S-XhoI-R: 5′-TCCACGCGTCTCGAGTTACAGTAAGGCTATCT-3′.

### Antibodies and reagents

Antibodies were obtained from the following sources; mouse anti-Nestin antibody (BD Transduction Laboratories), mouse anti-Synaptophysin (7H12, Cell Signaling Technology), rabbit anti-Map2 (D5G1, Cell Signaling Technology), anti-Cleaved Caspase-3 (Ab-2, Millipore-Sigma Aldrich), anti-PUMAα/β Antibody (G-3, Santa Cruz Biotechnology, Inc.), anti-Bcl-2 (C-2, Santa Cruz Biotechnology, Inc.), anti-Mcl-1 (S-19, Santa Cruz Biotechnology, Inc.), anti-Bax (N-20, Santa Cruz Biotechnology, Inc.), anti-Bad (Cell Signaling Technology), anti-SRSF1 (3G268, Santa Cruz Biotechnology, Inc.), anti-Tubulin (Millipore-Sigma-Aldrich). Mammalian protease inhibitors were obtained from Sigma-Aldrich (St Louis, MO). Bradford reagent was from BioWorld (Dublin, OH). MTT (3-(4,5-Dimethylthiazol-2-yl)-2,5-Diphenyltetrazolium Bromide) was obtained from Thermo Fisher Scientific (#M6494).

### Immunocytochemistry

hNSPs, hNPCs, immature neurons, and mature neurons were cultured in 2-well chamber slides (2 × 10^5^ cells/well). Cells were either exposed to 50 mM EtOH or left untreated. Immunocytochemistry was performed as described previously^24^. Briefly, at 24 h post-exposures, cells were fixed, blocked with 10% BSA in PBS for two hours, and incubated with primary antibodies (1:200 dilution in 5% BSA overnight at 4 °C with gentle rocking). Cells were washed and incubated with a secondary rhodamine or FITC antibodies (1:500 dilution in 5% BSA). Wells were then washed with PBS and mounted with Vectashield mounting solution containing DAPI. Then glass coverslips were added before imaging on a KEYENCE Fluorescence Microscope. Following primary antibodies were used in various experiments: Mouse anti-Nestin antibody (BD Transduction Laboratories), mouse anti-Synaptophysin (7H12, Cell Signaling Technology), rabbit anti-Map2 (D5G1, Cell Signaling Technology), anti-Cleaved Caspase-3 (Asp175, Cell Signaling Technology). Cleaved caspase-3 signal from red fluorescein positive cells was measured using a Batch Fluorescent Analysis and reported as the “average Cl-caspase-3-positive area (μm^2^)” over total area. The software used for the analysis was BZ-X Analyzer, provided with the KEYENCE Fluorescence Microscope.

### Multielectrode arrays (MEAs)

The MEA recordings were performed using the MEA-1060 system (Multichannel Systems, Reutlingen, BW, Germany). Before plating the cells, MEA dishes were sterilized by applying 70% ethanol and exposing to UV light for 120 min. Poly-D-lysine was used to hydrophilize and coat the surface of the MEA dishes. Laminin (Invitrogen/Thermo Fisher, Inc., Waltham, MA, USA) was also applied to the MEA surface (overnight at 37 °C) to promote cellular adhesion (to culture cells for > 10 days) and to increase the neural processes development. Human fetal brain cells were plated on MEAs (5000 cells/mm)^2^. After preparation, the suspended cells were placed onto the MEA dishes, settled, and adhered to the array within 4 h. Neurons were maintained and fed using an appropriate medium every 3 days with half media changes. MEA recordings were started when the cultures were 1, 3, and 4 weeks old. After placing the MEAs on the amplifier, recordings were performed using the MC Rack software (Reutlingen, BW, Germany) at a sampling frequency of 2000 kHz.

### MTT cell viability assay

hNSPs, hNPCs, primary human immature and mature neurons were cultured in 12-well tissue culture plates and treated in triplicate with different concentrations of EtOH (10 mM, 25mM, 50 mM and 75 mM) for 6, 24 and 48 h. After treatments, cells were incubated for 2 h at 37 °C with 150 µl of MTT (3-(4 5-dimethylthiazol-2-yl)-2 5-diphenyltetrazolium bromide) at 0.5 mg/ml working solution. The converted insoluble purple formazan was solubilized with 500 µl of acidic isopropanol (0.004 M HCl in isopropanol). Absorbance of the converted formazan was measured at a wavelength of 570 nm with a background subtraction at 650 nm. hNPCs were plated in 12-well tissue culture plates and transfected with 1 or 2 µg of pcDNA3.1-Mcl-1L or pcDNA3.1-Mcl-1S. Total DNA concentrations were adjusted with empty vector pcDNA3.1 per transfection. 24 h after transfections, cells were treated in absence or presence of EtOH 50 mM for 24 h and then MTT viability assays were performed as described above.

### Semi-quantitative RT-PCR

hNSPs, hNPCs, immature neurons, and mature neurons were exposed to 50 mM EtOH. At 6 and 24 h post-exposure time points, cells were harvested, and total RNA was extracted with an RNA extraction kit (New England Biolabs) according to the manufacturer's instructions. RT-PCR reactions were performed as described previously^24^. After treatment with DNase I, followed by phenol/chloroform extraction and EtOH precipitation, cDNAs were synthesized from total RNA using M-MuLV reverse transcriptase. RNA templates were removed by RNase H digestion. A total of 100 ng cDNA was used as template for PCR reactions. Alternatively spliced isoforms of Mcl-1 were amplified by PCR using Mcl-1F: 5′- GGACACAAAGCCAATGGGCAGGT-3′ and Mcl-1R: 5′-GCAAAAGCCAGCAGCACATTCCTGA-3′. β-Actin mRNA was also amplified from the same set of RNA samples by RT-PCR as an internal control using F: 5′-CTACAATGAGCTGCGTGTGGC-3′ and R: 5′-CAGGTCCAGACGCAGGATGGC-3′. Amplified gene products were resolved on a 1.5% DNA-agarose gel.

### Real-time qRT-PCR analysis of hMcl-1L and hMcl-1S isoforms

In order to analyze the ratio between human Mcl-1L and Mcl-1S isoforms, a quantitative RT-PCR was performed. hMcl-1L isoform coding region is composed of 3 exons: Exon 1, Exon 2 and Exon 3, while hMcl-1S isoform coding region is composed of 2 exons: Exon 1 and Exon 3. Two different probes, with fluorophore with different excitation/emission spectrum, were designed to anneal specifically the hMcl-1L or the hMcl-1S isoform, in correspondence of exons junction. hMcl-1L isoform probe anneals within the junction between Exon 2 and Exon 3, while hMcl-1S isoform probe anneals within the junction between Exon 1 and Exon 3. hMcl-1L isoform probe has FAM as fluorophore at 5′ and it is the following: 5′-CAAAGAGGCTGGGATGGGTTTGTG-3′. hMcl-1S isoform probe has HEX as fluorophore at 5′ and its sequence is: 5′-CGGCCTTCCAAGGATGGGTTTGTG-3′. The RT-qPCR was performed in LightCycler 480 (Roche Applied Science) using Luna® Universal Probe One-Step RT-qPCR Kit (New England Biolabs). Briefly, total RNA of treated cells was isolated using the TRIzol™ Reagent protocol. 100 ng of RNA was used as template. The RT-qPCR reaction mixtures contained: 1 × Luna Universal Probe One-Step Reaction Mix, 1 × Luna WarmStart RT Enzyme Mix, 400 nM of each primer, 400 nM of each probe, template RNA and nuclease-free water to a final volume of 20 μL. The RT-qPCR protocol was: reverse transcription at 55 °C for 10 min, initial denaturation at 95 °C for 1 min, then 45 cycles of denaturation at 95 °C for 15 s and extensions at 60 °C for 1 min with single acquisition. The primers used were designed to anneal both isoforms within Exon 1 (Forward) and Exon 3 (Reverse) and are the following: H1-hMcl-1-F: 5′-GGACACAAAGCCAATGGGCAGGT-3′ and H3-hMcl-1-R: 5′-GCAAAAGCCAGCAGCACATTCCTGA-3′. pcDNA3.1-Mcl-1L and pcDNA3.1-Mcl-1S were used as standards and controls for specificity of the probes.

### Droplet Digital Polymerase Chain Reaction (ddPCR)

In this study, the QX200™ Droplet Digital™ PCR System (Bio-Rad, Hercules, CA, USA) was used. The ddPCR reaction mixtures contained: 1 × ddPCR™ Supermix for Probes (No dUTP) (Bio-Rad, Pleasanton, CA, USA), 500 nM of each primer, 500 nM of each probe (FAM and HEX), sample and water to a final volume of 22 μL. Droplets were generated using the QX200™ Droplet Generator (DG). A DG8™ cartridge holder and gasket with 70 μL of Droplet Generation Oil/well, 20 μL of PCR reaction mixture were used. From the DG8 cartridge, 40 μL of the generated droplets were transferred to ddPCR™ 96-well-plate. The plate was then heat-sealed using a PX1™ PCR plate sealer and a pierceable foil seal. PCR was performed using a C1000 Touch™ deep-well thermal cycler. The thermocycling protocol was: initial denaturation at 95 °C for 10 min, then 40 cycles of denaturation at 95 °C for 30 sec, annealing at 55 °C for 1 min and extension for 1 min, followed by a final last incubation at 98 °C for 10 min and storage at 4 °C. After amplification, the ddPCR™ 96-well-plate was placed into a plate holder into the QX200™ Droplet Reader. PCR-positive and PCR-negative droplets of each sample were analyzed and fluorescent signals of each droplet were counted and quantified.

### Statistical analysis

All of the values presented on the graphs are given as means ± S.E.M. ANOVA and unpaired Student’s *t* tests were used to analyze the statistical significance, and *P* values < 0.05 were considered statistically significant.
